# Patient-Specific 3D-Printed Miniplates for Free Flap Fixation at the Mandible: A Feasibility Study

**DOI:** 10.3389/fsurg.2022.778371

**Published:** 2022-03-14

**Authors:** Kilian Kreutzer, Claudius Steffen, Steffen Koerdt, Christian Doll, Tobias Ebker, Susanne Nahles, Tabea Flügge, Max Heiland, Benedicta Beck-Broichsitter, Carsten Rendenbach

**Affiliations:** Department of Oral and Maxillofacial Surgery, Charité - Universitätsmedizin Berlin, corporate member of Freie Universität Berlin and Humboldt-Universität zu Berlin, Berlin, Germany

**Keywords:** computer aided design/manufacture (CAD/CAM), mandible reconstruction, miniplate, fibula free flap, patient-specific, outcome

## Abstract

**Background:**

This study was conducted to evaluate the feasibility, clinical outcomes, and accuracy of patient-specific 3D-printed miniplates for mandible reconstruction with fibula free flaps.

**Methods:**

A feasibility study was conducted with 8 patients. Following virtual planning, patient-specific 1.0 mm titanium non-locking miniplates were produced via laser selective melting. 3D-printed cutting and drilling guides were used for segmental mandible resection and flap harvesting. Flap fixation was performed with two 4-hole miniplates and 2.0 mm non-locking screws (screw length 7 mm) for each intersegmental gap. Clinical follow-up was at least 6 months. Preoperative and postoperative CT/cone beam CT data were used for 3D accuracy analysis and evaluation of bone healing. Plate-related complications were monitored clinically.

**Results:**

Patient-specific miniplate fixation of all flaps was successfully conducted (4 mono-segmental, 4 dual-segmental) with high accuracy (3.64 ± 1.18 mm) between the virtual plan and postoperative result. No technical complications were encountered intraoperatively. Osseous union occurred in all intersegmental gaps (1 partial, 18 complete) after 10 ± 2 months. No material fracture, dislocation, or plate exposure was observed.

**Conclusions:**

Based on this pilot observational study including a limited number of patients, free flap fixation for mandibular reconstruction with patient-specific 3D-printed miniplates is feasible and associated with high accuracy, bone healing, and remote soft tissue complications.

## Introduction

**Figure 1:**
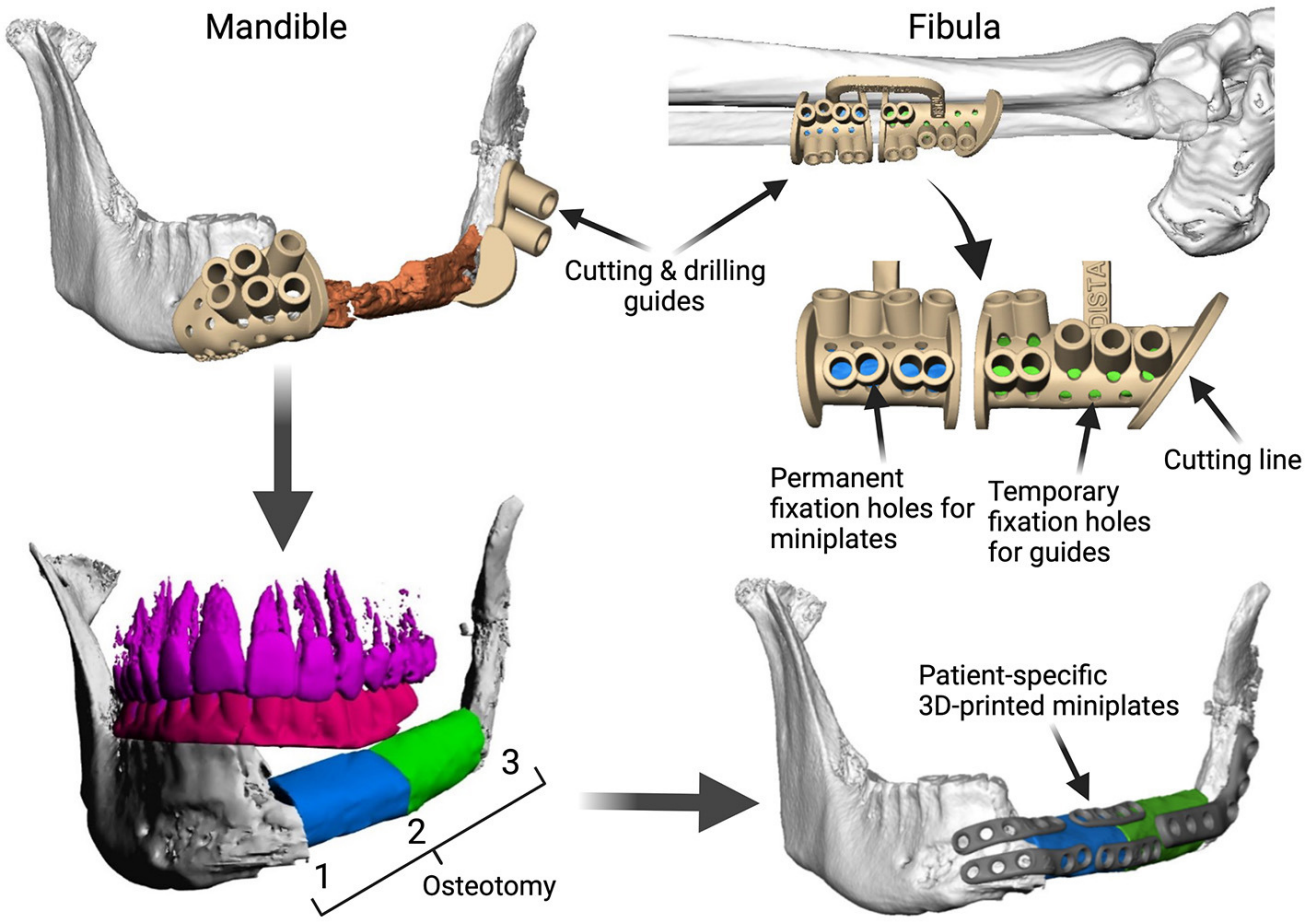
Demonstration of the CAD/CAM-workflow: Cutting and drilling guides placed at the mandible defect (orange) and the fibula segments (green and blue). Guides include holes for temporary guide fixation as well as permanent holes for miniplates fixation. Bottom left: Visualization of the dentition of the upper jaw (violet) and dental setup of the lower dentition (pink) for planning of the dental implant insertion. Osteotomy lines 1, 2, and 3 are also shown. Final reconstruction results with patient-specific 3D-printed miniplates are demonstrated in the bottom right corner. Created with BioRender.com.

Fibula free flap (FFF) reconstruction is the treatment of choice for segmental mandibular defects. Either load-bearing (locking) reconstruction plates with bi-cortical screws or load-sharing miniplates with mono-cortical non-locking screws are used for flap fixation. Following the introduction of computer-generated models in the early 1990s (1), virtual planning was first described for mandible reconstruction in 2005 (2). The combination of the computer-aided design/computer aided manufacturing (CAD/CAM) workflow with milled and, later, selective laser-melted patient-specific reconstruction plates revolutionized mandible reconstruction with free flaps (3). The main advantages of patient-specific 3D-printed plates are high surgical precision, efficiency, and decreased surgery time (4, 5). Higher mechanical integrity due to differences in plate design, increased bone-plate contact area, and most importantly a lack of predetermined breaking points from manual bending were also demonstrated recently (6, 7). Today, a backward-planned, fully guided CAD/CAM procedure is the state of the art, with a good aesthetic and functional outcome (Figure 1).

However, plate-related complications are still common when using patient-specific reconstruction plates and do not only occur in patients with relevant risk factors like radiotherapy or multi-segmental defects (7–9). Complications also include soft tissue complications and pseudarthrosis, which are frequently observed within the first postoperative year (7). In comparison to low-profile miniplates, 2.0–3.0 mm reconstruction plates increase local compression on the surrounding tissues, which potentially favors the onset of plate exposure. Their increased stiffness reduces intersegmental movements between flap segments and between the fibula and mandible to a critically low range (10). This is unfavorable and may increase risk for pseudarthrosis, since certain amounts of micromovement and microstrain are essential for sufficient bone healing (11, 12).

In contrast, load-sharing miniplates transmit applied loads through both the plate and the bone, which stimulates bone healing under frictional contact between the bone segments (13). From a mechanobiological point of view, the use of miniplates is therefore beneficial. Additionally, plate removal due to plate-related complications or in the course of preparing the patient for dental implantation and, later, prosthetic rehabilitation (14) is easier with miniplates and reduces the risk of damaging the vascular pedicle or marginal mandibular branch of the facial nerve (15). The use of conventional miniplates for free flap fixation has been described several times (16, 17). However, complications also occur with conventional miniplates (18), and plate fractures are registered more frequently (8). This is due to pre-determined breaking points during manual bending with forceps and potential poor anatomical reduction of the (joint-carrying) segments, setting the fixation under unfavorable pre-stress (19).

Considering the well-known advantages of the CAD/CAM workflow and patient-specific plates in general (efficiency, handling, no predetermined breaking points) together with the advantages of miniplates (mechanobiology, plate removal, soft tissue management), it was hypothesized that the combination of both worlds could significantly improve the clinical outcome after mandible reconstruction with free flaps. Accordingly, this feasibility study aimed to be the first to evaluate patient-specific 3D-printed miniplates for free flap fixation.

## Materials and Methods

Ethical approval was obtained by the local ethics committee (EA2/138/18). The cases represent the first clinical use of patient-specific 3D-printed miniplates for mandible reconstruction. The inclusion period was between July and November 2019. Only patients planned for segmental mandibular resection due to malignant or benign tumors or osteoradionecrosis and primary reconstruction with a mono- or dual-segmental fibula free flap were included. A minimum clinical and radiological postoperative follow-up period of 6 months was required. Follow-up analysis was performed until December 2020. Multisegmented fibula free flaps (>2 segments) and bruxism were exclusion criteria.

The CAD/CAM planning procedure followed previously reported workflow (20): All patients underwent preoperative CT/cone beam CT (CBCT) scans of the mandible and CT angiography of the lower extremities to check the perfusion status and to provide 3D data for virtual planning. In patients with malignancies, the treatment protocol was defined after staging completion in an interdisciplinary tumor conference.

Tomographic imaging data of both the donor and recipient sites were uploaded to cloud-based software (IPS Gate, KLS Martin Group, Mühlheim, Germany). Resection margins were defined according to the radiological and clinical findings by a senior consultant of the Department of Oral and Maxillofacial Surgery, Charité – University Medicine, Berlin (Germany). Flap reconstruction and the design of cutting and drilling guides and plates were virtually performed in web meetings with engineers from the manufacturer and a senior consultant (KLS Martin Group, Mühlheim, Germany). The plate thickness was 1.0 mm, the screw diameter was 2.0 mm, and the screw length was 7.0 mm. As with patient-specific 3D-printed reconstruction plates, a linear plate design was chosen to increase fatigue strength between the plate holes. Each intersegmental gap was bridged with two 4-hole miniplates on the vestibular side. The placement of the 3D-printed miniplates was in accordance with the biomechanical tension lines in fracture treatment. The design of some plates was modified to preserve the function of the mental nerve. Cutting and drilling guides were 3D printed. 3D-printed miniplates were manufactured by a laser-melting procedure.

In patient 7, a combined approach with anterior miniplates and a posterior reconstruction plate was performed. This was because a previous fibula free flap had been transplanted and needed surgical revision and a second FFF due to osteoradionecrosis. A posterior reconstruction plate was chosen due to the small dimensions of the previous flap.

Resection, flap harvesting, and reconstruction with miniplates were performed in a 2-team approach by senior consultants. Flap harvesting was performed using the lateral approach. A piezoelectric bone-cutting device was used for all osteotomies after guide fixation (Piezosurgery, Mectron, Cologne, Germany). Monocortical 2.0 mm non-locking screws with a length of 7.0 mm were used for plate fixation. Prolene 8.0 single-button sutures were used for both arterial and venous anastomosis. Alternatively, a coupler system was used for venous anastomosis. Intraoral wound closure was performed with Vicryl 3-0. Subcutaneous Fraxiparin^®^ 0.3 mL was administered twice a day for 1 week. Initial nutrition was ensured with a nasogastric tube. A protective tracheostomy was performed in all patients until postoperative swelling was reduced and patients were able to swallow. Flap perfusion was controlled closely during the first 7 days. After discharge, outpatient controls with intra- and extraoral examination were performed on a regular basis postoperatively, including at least 1 CT or CBCT control. In clinical examinations particular attention was paid to soft tissue complications, material failure, or osseous complications.

### 3D Accuracy

**Figure 2:**
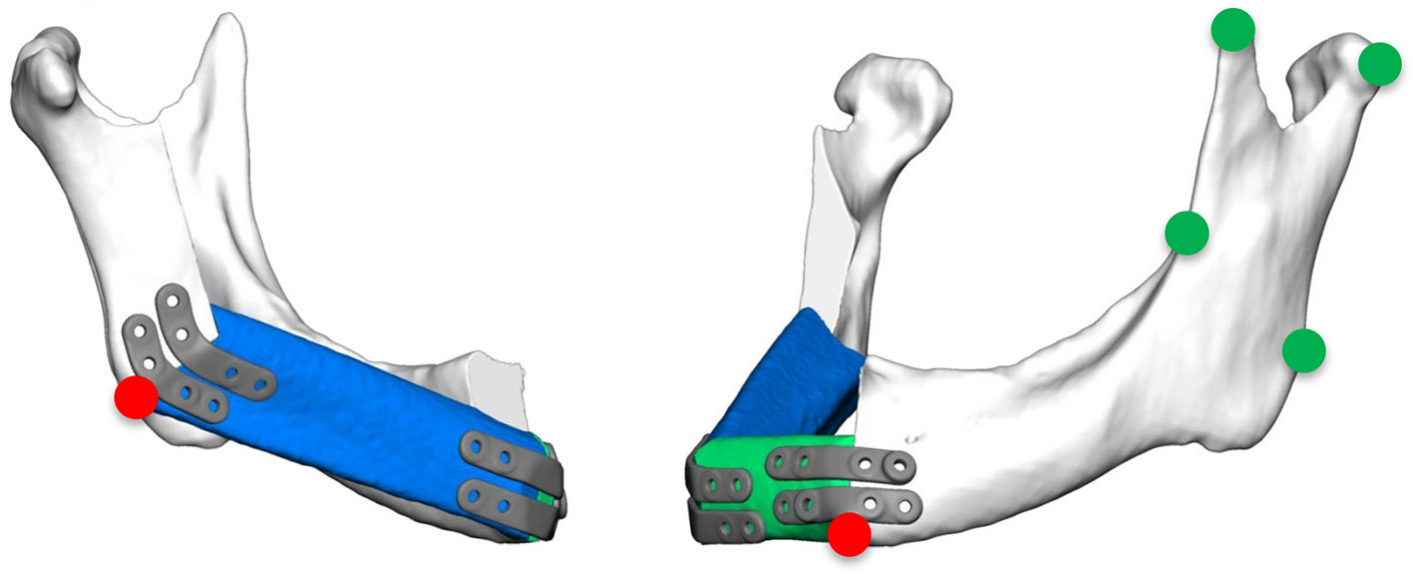
Green marks: measurement points at the contralateral ascending ramus to evaluate the quality of fitting; red marks: measurement points at the base of the mandible at the connection of the original bone to the FFF and the most inferior point of the ipsilateral incisura semilunaris.

Accuracy measurements were performed by a single independent investigator (CS) with the 3D GOM software (GOM, Braunschweig, Germany). After semi-automatic alignment of the CAD/CAM planning and the postoperative scan, which was based on the non-operated side of the mandible, the quality of fitting was determined as the mean deviation of 4 reproducible points at the contralateral ascending ramus by calculating the vector magnitude (Figure 2; green marks). The vector magnitude was also used for all other values in the precision analysis. For further analysis of accuracy measurements, care was taken to select points in the reconstruction region that could be reliably identified in all images, undisturbed by metal imaging artifacts. The deviation (planning vs. postoperative scan) of the medial and distal basal contact points at the transition of the transplant to the local bone were measured to evaluate accuracy. In addition, the most caudal point of the ipsilateral incisura semilunaris was measured to reflect the positioning of the ipsilateral ascending ramus (Figure 2; red marks). The mean of all three values (mesial gap, distal gap, ipsilateral incisura semilunaris) was determined. By subtracting the quality of fitting the corrected mean deviation was calculated. This resulted in a value demonstrating the absolute deviation (21).

All data were included in a database (Microsoft Excel, Microsoft Corporation, Redmond, USA). Descriptive statistics were performed using Microsoft Excel.

### Osseous Union

The degree of osseous consolidation was determined by 2 independent observers based on postoperative sectional imaging (CT or CBCT). For each osteotomy line, the highest degree of osseous consolidation was classified according to the axial view as previously published by Yla-Kotola et al. (22). Non-union was classified as a lack of apposition of the bone segments on every section of the mandible. Partial-union was determined as incomplete bone fusion in every section of the mandible. And complete union was valued as good bone fusion of the osteotomized sections of the fibula and the neomandible. This led to 2 values in single-segment reconstructions and 3 values in 2-segment transplants. Osteotomy 1 always represented the osteotomy line at the angle of the mandible (ipsilateral posterior), osteotomy 2 the ipsilateral anterior, and osteotomy 3 the contralateral osteotomy. In patient 7, the posterior gap was not quantified, as it was bridged with a reconstruction plate.

## Results

**Figure 3:**
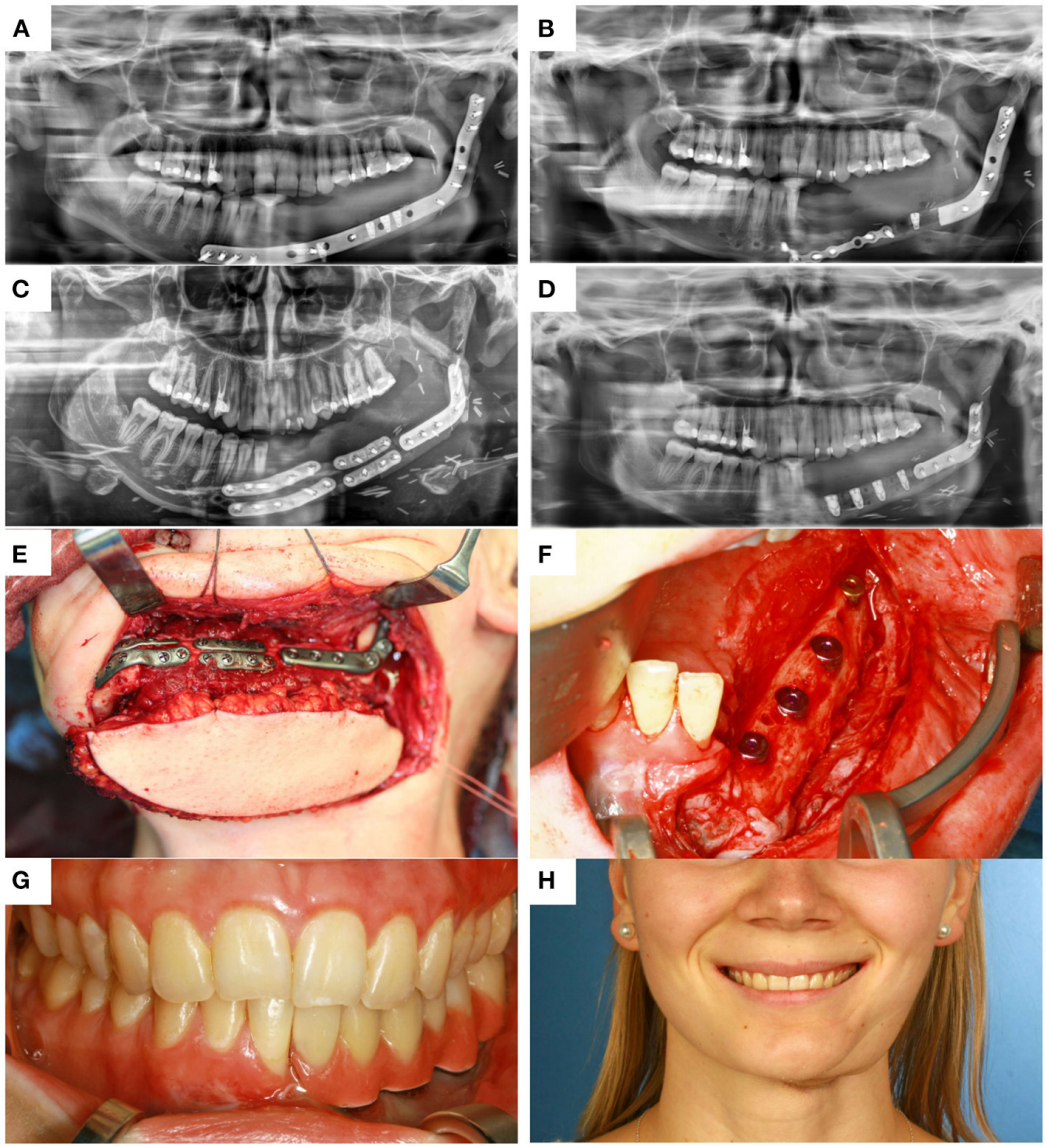
**(A)** control after placement of dental implants into the first CAD/CAM FFF with reconstruction plate, **(B)** partial loss of the FFF and partial removal of the reconstruction plate with re-osteosynthesis, **(C)** control after mandible reconstruction with the second CAD/CAM FFF with miniplates and small reconstruction plate, **(D)** control after intraoral removal of the miniplates and simultaneous dental implant insertion, **(E)** intraoperative site of **(C)**, **(F)** intraoperative site of **(D)**, **(G)** dental rehabilitation with implant supported fixed prosthesis **(H)** clinical impression 14 months postoperatively.

Eight patients (3 men and 5 women, mean age 63 ± 19 years; range 18–83 years) underwent mandible reconstruction with a fibula free flap and patient-specific 3D-printed miniplates between July and November 2019. Table 1 illustrates the patient characteristics. Certain pre-existing diseases considered were diabetes (*n* = 2), osteoporosis (*n* = 0), and vascular diseases (*n* = 1). Seven patients were treated due to primary oral squamous cell carcinoma (87.5%). One patient required a second fibula flap owing to osteoradionecrosis (ORN) after recent fibula reconstruction due to the resection of an Ewing‘s osteosarcoma (12.5%). Four patients received a mono-segmental and 4 patients a dual-segmental fibula flap. The mean flap length was 63 mm (SD ± 12 mm). Intraoral wound closure was performed with tibialis posterior muscle (*n* = 2), posterior septum (*n* = 2), or a skin island (*n* = 4). Screw insertion and plate fixation following pre-drilling with cutting and drilling guides was unproblematic, without the need for additional drilling due to imperfect fitting. Four patients (50%) received adjuvant radio(chemo)therapy. In this study, no revisional surgery was necessary, and no flap was either partially or completely lost. Radiological follow-up was performed 10 ± 2 months after surgery. Concerning patients with squamous cell carcinoma as initial diagnosis, there were no locoregional recurrences or distant metastases noted within the follow-up. Seven patients presented a clinically uneventful course regarding infections, wound healing disorders, plate exposure, and material failure. In patient 2, a late-onset infection with fistula in the canine region was registered after 4 months. Surgical exploration revealed partial screw loosening but complete osseous union of the former anterior intersegmental gap. Early plate removal in this region was therefore performed during the same procedure. In patient 7, all miniplates were removed as early as 7 months after surgery, as complete osseous union was detected in a postoperative CBCT, and the patient had advocated for short-term dental rehabilitation. Prosthetic rehabilitation was completed 13 months after reconstructive surgery in this patient, with an excellent functional and aesthetic result (Figure 3).

**Table 1:** Patient characteristics; SCC, squamous cell carcinoma; ORN, osteonecrosis/late-onset flap loss; R(C)T, radio(chemo)therapy.

**No**	**Sex**	**Age**	**Indication**	**R(C)T**	**Segments**	**Length [mm]**	**Defect**	**Planning illustration**
1	F	83	SCC	No	1	60	L	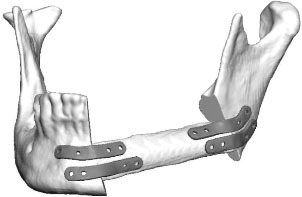
2	F	70	SCC	No	1	59	L	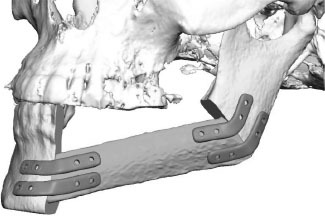
3	F	77	SCC	Adjuvant RT	1	45	L	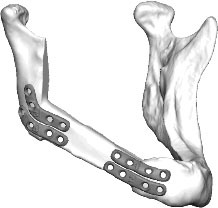
4	M	51	SCC	No	2	80	CL	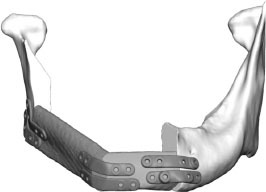
5	M	66	SCC	Recent RCT	2	74	L	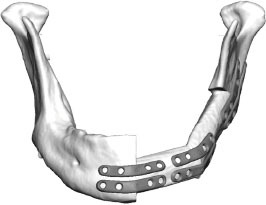
6	F	64	SCC	Adjuvant RT	2	77	CL	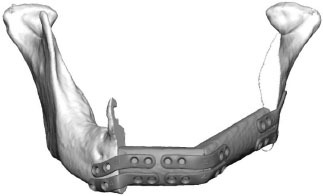
7	F	18	ORN	Recent RCT	2	58	L	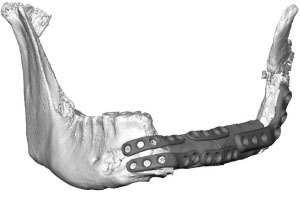
8	M	76	SCC	No	1	54	L	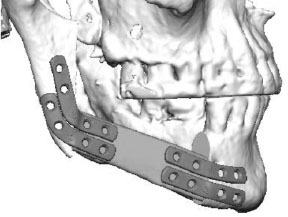

Precision analysis revealed that the planning could be superimposed with the postoperative imaging with high accuracy (Table 2). The values for quality of fitting were between 0.19 mm and 1.53 mm (mean value 0.7 mm). The planned reconstruction was achieved with a heterogeneous deviation between 0.82 mm and 9.72 mm. The corrected mean deviation over all marks and patients was 3.64 mm (SD ± 1.18 mm). Mean deviations in 2-segment reconstructions were found to be only slightly higher than in 1-segment reconstructions (3.84 mm vs. 3.44 mm). In the radiological assessment of intersegmental regions, only 1 gap was classified as a partial union, while all other gaps were defined as complete osseous unions (Table 3).

**Table 2:** Precision Analysis.

**Patient No**.	**1**	**2**	**3**	**4**	**5**	**6**	**7**	**8**
Mesial gap [mm]	1.89	3.76	4.43	2.51	2.82	2.60	3.07	3.26
Distal gap [mm]	6.04	2.90	4.48	6.75	5.48	5.90	3.14	6.48
Incisura semilunaris [mm]	5.15	0.82	5.02	9.72	3.03	7.75	2.92	4.24
**Mean deviation [mm]**	**4.36**	**2.49**	**4.64**	**6.32**	**3.78**	**5.42**	**3.04**	**4.66**
Quality of fitting [mm]	1.37	0.27	0.56	0.76	1.53	0.60	0.31	0.19
**Mean deviation corrected [mm]**	**2.99**	**2.22**	**4.08**	**5.56**	**2.25**	**4.82**	**2.73**	**4.47**

*Deviation = difference between preoperative planning and postoperative precision*.

**Table 3:** Qualitative analysis of osseous union (1 = non-union, 2 = partial-union, 3 = union).

**Patient No**.	**1**	**2**	**3**	**4**	**5**	**6**	**7**	**8**
Osteotomy 1	3/3	3/3	3/3	3/3	3/3	3/3	X	3/3
Osteotomy 2	3/3	3/3	3/3	3/3	3/3	3/3	3/3	3/3
Osteotomy 3	X	X	X	3/3	3/2	3/3	3/3	X

## Discussion

Plate-related complications after mandible reconstruction with free flaps are common, and neither conventional and patient-specific reconstruction plates nor conventional miniplates can be considered as ideal fixation systems given the specific needs of patients undergoing this procedure. We conducted a study to examine the feasibility of newly designed patient-specific 3D-printed miniplates.

The precise implementation of preoperative planning represents the greatest advantage of the CAD/CAM technology. Accuracy plays a prominent role in the reconstruction of the lower jaw, as it directly influences the restoration or maintenance of the occlusion and the pitch of the temporomandibular joints, and thus function and aesthetics. In this study, the preoperative VSP (virtual surgical planning) was compared to the postoperative result. With a corrected mean deviation of 3.64 mm over all marks and patients, a high level of accuracy was achieved with 3D-printed miniplates. Comparison of accuracy measurements is difficult, since a wide range of measurement techniques has been described for VSP mandible reconstruction (23–31), and the results also depend on the defect. In a systematic review, van Baar et al. identified several reasons that postoperative results never fully match virtual plans, including image acquisition, segmentation, 3D printing, surgery, and evaluation (26). Among 42 studies, they found deviations ranging from 0 to 12.5 mm, demonstrating that computer assisted mandible reconstruction is superior to conventional plating techniques, with 2.81–6.35 mm greater deviations of the condyle in the conventional plating group. The results of the current study are at the upper end of this scale. Its high accuracy is further underlined by the fact that no malocclusion was found in any of the patients. Comparison of our study with the review by van Baar et al. needs to be performed cautiously due to different analyzation method and software.

The high precision achieved by the CAD/CAM technology may positively affect development of pseudarthrosis. Several factors influence bone healing, both in general and after free flap reconstruction of the mandible. Bone healing will not occur when bone segments are mal-positioned and no adequate contact area exists. Osseous union at the osteotomy site after free flap reconstruction is indicative of successful flap surgery with high precision (32). There is no standardized procedure for evaluating the ossification in the osteotomy gap (22, 32–34). This is even more challenging because there is no radiological definition of a “healed” fracture (35). The inevitable superimposition of artifacts due to the adjacent osteosynthesis significantly impairs quantitative assessment.

However, as far as can be concluded from the non-quantitative analysis with 8 patients, the high rate of full osseous unions in this pilot study must be outlined. Recently, it was demonstrated that partial osseous union and pseudarthrosis occur much more frequently under fixation with conventional and especially rigid patient-specific 3D-printed reconstruction plates (7, 16, 17, 34). There is a lack of prospective randomized controlled studies comparing load-bearing rigid and load-sharing miniplate fixation for mandible reconstruction, but non-union with miniplates seems less frequent (17, 36, 37). While previous and adjuvant radiotherapy, chemotherapy, and the number of segments were recently seen to be associated with impaired bone healing, differences between fixation techniques may be explained by fundamentally different biomechanical principles. The load distribution in a load-sharing osteosynthesis can lead to improved bone consolidation if the bone fragments are well-adapted. Stress shielding, as provoked by stiff reconstruction plates, is a well-known cause of a lack of consolidation due to insufficient stimulation in the osteotomy gaps (11, 12, 17, 38, 39). This is in accordance with recent findings from *in vitro* biomechanical analyses (6, 10), which revealed that miniplate fixation allows for more intersegmental micromovements than conventional and patient-specific 3D-printed reconstruction plates. However, *in vivo* evidence for this hypothesis in the context of mandible and free flap mandibular reconstruction is still missing.

Within the scope of this investigation, only 1 patient suffered a soft tissue complication, namely plate-related infection. Remarkably, no plate exposure occurred. Others found plate extrusion rates of 6–14% with miniplates, 8–24% with reconstruction plates, and 29% for CAD/CAM locking reconstruction plates (7, 16, 17). Despite this, however, no study has reported a significant difference between fixation techniques. Also from this study no general statement concerning possibly lower complication rates can be made—the patient number is too small and follow-up is too short. In terms of feasibility of patient-specific 3D-printed miniplates it can be concluded that osteosynthesis using these type of plates seems to be reliable.

Regarding soft tissue management and the further clinical course, the possibility to remove miniplates at a later stage via an intraoral approach when they have been inserted using wide exposure for segmental resection from an extraoral position represents another important advantage of miniplate fixation. The complete removal of reconstruction plates, if necessary, is usually only feasible via a laborious submandibular approach. In contrast, an intraoral approach, potentially combining plate removal with dental implant insertion, enables a rapid and less stressful procedure and can be conducted in an outpatient setting. Another, so far hardly discussed, factor is the lack of compatibility of a reconstruction plate with dental rehabilitation. In patient 7, the reconstruction plate from prior mandible reconstruction was the limiting factor for both the implant position and peri-implant soft tissue management (Figure 3A). Before this patient received the second flap with miniplates, there was a local infection and, consequently, loss of the implant-bearing part of the fibula due to poor soft tissue management over the residual part of the reconstruction plate (Figure 3B). Certainly, backward-planning including positioning of dental implants is possible before surgery (40), but reconstruction plates often impede reliable soft tissue management for dental implants (20).

As for the CAD/CAM workflow in general (41), shifting treatment time from the operating room to the preoperative VSP is another advantage of flap fixation with patient-specific miniplates. In comparison to 3D-printed reconstruction plates we do not see a disadvantage concerning time consumption with 3D-printed miniplates due to the possibility to adapt miniplates to the fibula bone in a two team approach before isolating the fibula free flap from the leg. In comparison to conventional miniplates the advantage of the CAD/CAM workflow is even more important than with reconstruction plates, considering the more complex intraoperative plate bending required for sufficient anatomical repositioning.

The limitations of this study include the small number of patients, the non-quantitative analysis of bone healing, the observational (feasibility) study design, and the lack of a control group. Our strict exclusion criteria (more than 2 segments of fibula graft and bruxism) due to reasons of safety also limit the meaningfulness to patients with more complex situations. Accordingly, neither a clear-cut recommendation to use patient-specific 3D-printed miniplates instead of patient-specific reconstruction plates nor an evidence-based demonstration of the superiority of such miniplates over conventional fixation plates can be provided. Yet, the results from this study are encouraging.

In summary, fibula free flap fixation with patient-specific 3D-printed miniplates is technically feasible, can be conducted with high accuracy, and is associated with minimal plate-related complication rates and a good chance of bone healing in the osteotomy gaps (Table 4). Following this pilot, prospective randomized controlled trials comparing the clinical outcomes and complication rates of a) conventional versus patient-specific miniplates and b) patient-specific mini- and reconstruction plates are necessary.

**Table 4:** Advantages of 3D-printed miniplates.

**Advantages of CAD/CAM planning**	**Advantages of miniplates**
Virtual planning	Load sharing fixation
Backward planning	Hardware removal from intraorally
Greater precision of the reconstruction	Partial hardware removal possible
Shorter surgery	Lower thickness of the osteosynthesis plates
↓↓	
patient-specific 3D-printed miniplates	

## Data Availability Statement

The original contributions presented in the study are included in the article. Further inquiries can be directed to the corresponding author.

## Ethics Statement

The studies involving human participants were reviewed and approved by Charité - Universitätsmedizin Berlin. The patients/participants provided their written informed consent to participate in this study. Written informed consent was obtained from the individual(s) for the publication of any potentially identifiable images or data included in this article.

## Author Contributions

KK and CR: conceived and designed the analysis, performed the analysis, and drafted this manuscript. CS: collected the data, performed the analysis, and drafted this manuscript. SK and CD: collected the data and drafted this manuscript. TE, SN, and TF: drafted this manuscript. MH and BB-B: conceived and designed the analysis and drafted this manuscript. All authors contributed to the article and approved the submitted version.

## Conflict of Interest

MH received speaker remuneration by Karl Leibinger Medizintechnik GmbH and Co KG. CR received research funding for other projects by Karl Leibinger Medizintechnik GmbH and Co KG. The remaining authors declare that the research was conducted in the absence of any commercial or financial relationships that could be construed as a potential conflict of interest.

## Publisher's Note

All claims expressed in this article are solely those of the authors and do not necessarily represent those of their affiliated organizations, or those of the publisher, the editors and the reviewers. Any product that may be evaluated in this article, or claim that may be made by its manufacturer, is not guaranteed or endorsed by the publisher.
